# Supplementary material for: Complex‐centric proteome profiling by SEC‐SWATH‐MS
Source: Mol Syst Biol. 2019 Jan 14;15(1):e8438. doi: 10.15252/msb.20188438 (PMC6346213; doi:10.15252/msb.20188438)
Supplement: Supplementary file 6 — Dataset EV5 [file MSB-15-e8438-s006.zip › feature_plots_corum/809-2.pdf]

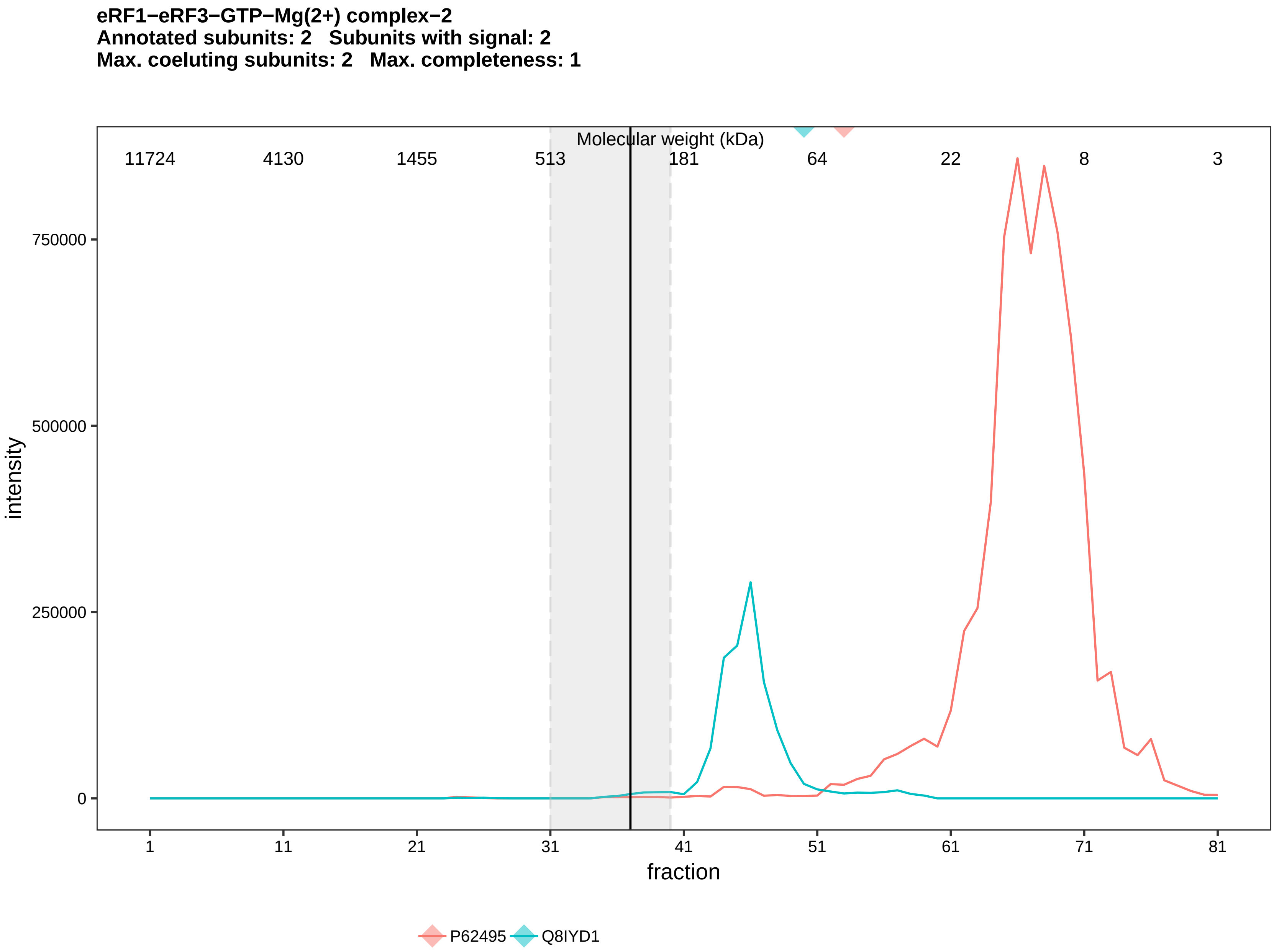
eRF1−eRF3−GTP−Mg(2+) complex−2
Annotated subunits: 2   Subunits with signal: 2
Max. coeluting subunits: 2   Max. completeness: 1
Molecular weight (kDa)
11724
4130
1455
513
181
64
22
8
3
750000
500000
250000
0
intensity
1
11
21
31
41
51
61
71
81
fraction
P62495
Q8IYD1
